# Sexual practices, sexual behavior and HIV risk profile of key populations in Nigeria

**DOI:** 10.1186/s12889-019-7553-z

**Published:** 2019-09-02

**Authors:** Bartholomew Ochonye, Morenike Oluwatoyin Folayan, Adesegun O. Fatusi, Bamidele M. Bello, Babatunde Ajidagba, Godwin Emmanuel, Paul Umoh, Ayo Yusuf, T. Jaiyebo

**Affiliations:** 1 Heartland Alliance International, Abuja, Nigeria; 2 New HIV Vaccine and Microbicide Advocacy Society, Lagos, Nigeria; 3 Department of Child Dental Health, Obafemi Awolowo University, Ile-Ife, Nigeria; 4 College Research and Partnership Advancement (CoRPA) Office, College of Health Sciences, Obafemi Awolowo University, Ile-Ife, Nigeria; 5 Population and Reproductive Health Programme, Obafemi Awolowo University, Ile-Ife, Nigeria; 6 Academy for Health Development (AHEAD), Ile-Ife, Nigeria

**Keywords:** Key populations, Men who have sex with men, Female sex workers, People who inject drugs, Sexual behavior, Sexual practices, Nigeria

## Abstract

**Background:**

There is little evidence on the need for differentiated HIV prevention services for men who have sex with men (MSM), female sex workers (FSW) and people who inject drugs (PWID in Nigeria. The aim of the study was to determine and compare the HIV sexual risk profiles of FSW, MSM and PWID resident in Nigeria; and identify factors associated with condom use among the groups. This will help identify if differentiated HIV prevention services are needed for MSM, FSW and PWID in Nigeria.

**Methods:**

This is a cross-sectional study. Data on sexual practices (anal, vaginal and oral sex), history of alcohol and psychoactive substance use, and high risk sexual behaviors for HIV infection (inconsistent use of condom) was collected from study FSW, MSM and PWID resident in Enugu, Nassarawa, Benue, and Akwa-Ibom States of Nigeria between April and June, 2015. Association between sexual practices, alcohol and psychoactive substance use, and HIV sexual risk behaviors; and differences in sexual risk behaviors of MSM, FSW and PWID were determined using Pearson chi-square for categorical variables, and t-test for continuous variables. Determinants of condom use in the last 30 days were identified using logistic regression analysis.

**Results:**

The study population consisted of 188 (38.5%) FSW, 145 (29.7%) MSM and 155 (31.8%) PWID. MSM (AOR: 0.17; 95%CI: 0.05–0.67; *p* = 0.01) and PWID (AOR: 0.07; 95%CI: 0.02–0.21; *p* < 0.001) were significantly less likely than FSW to have used condom in the last 30 days. A lower proportion of FSW and PWID used condom during anal sex in the last 12 months when compared with MSM (p < 0.001 respectively). The proportion of MSM (23.5%) and FSW (23.4%) who had ever used psychoactive drugs was high. Of those who had ever used psychoactive drugs, 25.0% of FSW and 29.4% of MSM had injected drugs in the last 30 days of the survey. Also, 39.3% of PWID shared needles and syringes. The use of psychoactive substances (AOR: 5.01; 95%CI: 2.59–9.68; *p* < 0.001) and the ability to negotiate condom use (AOR: 2.04; 95%CI: 1.06–3.93; *p* = 0.03) were factors associated with condom use in the last 30 days of the survey.

**Conclusion:**

HIV prevention programs designed for MSM, FSW and PWID need to address inconsistent condom use during sex by addressing condom negotation skills. This sexual risk behavior is common to the three groups.

**Electronic supplementary material:**

The online version of this article (10.1186/s12889-019-7553-z) contains supplementary material, which is available to authorized users.

## Background

Key populations –people who inject drugs (PWID), men who have sex with men (MSM) and female sex workers (FSW) – have higher vulnerability to HIV infection than the general population. The prevalence of HIV infection among PWID is 28 times higher than that of the general population; while the prevalence of HIV among MSM and FSW is estimated to be 19 and 12 times higher than that of the general population respectively, in low- and middle-income countries [1]. The HIV prevention and treatment needs of key populations have however, been inadequately addressed by the national HIV and AIDS responses in most low- and middle-income countries (LMICs), particularly in sub-Saharan Africa countries [2] one of which is Nigeria.

The HIV response needs of key populations in Nigeria is completely donor driven [3]. Yet the HIV response is critical to the success of achieving epidemic control in Nigeria because the HIV epidemic among key populations is a key driver of the national epidemic – there is a concentrated epidemic among key populations and another epidemic among the general population [4]. Strategic but distinct interventions are needed to reduce the HIV incidence in the general and key populations. The epidemic among the general population is largely driven by unprotected heterosexual intercourse and mother to child transmission of HIV infection [5]. It is speculated that the driver of the epidemic among key populations is unprotected sexual intercourse and untreated sexually transmitted infections [5]. The evidence for the driver of the HIV epidemic among key populations is largely anecdotal.

Strategic interventions were designed for key populations based on these anecdotal evidences. These proposed interventions are documented in key national documents such as the National HIV Strategic Plan [6] and the National HIV Prevention Plan [5]. Specific national guidelines for programming with FSW [7] and PWID [8] were also developed. To facilitate programming, a local epidemiological appraisal was conducted to estimate the size of FSW, MSM and PWID, and map ‘hot spots’ where key populations’ converge in large numbers [9]. Other specific interventions include the design and implementation of one stop-shops to provide services across the prevention-testing-treatment-care cascade for key populations in Nigeria [10]. The HIV prevention packages for MSM, FSW and PWID provide frequent access to HIV testing, condom access (including lubricants for MSM) and access to treatment of sexually transmitted infections. There is no harm-reduction program for PWID [5].

Despite these efforts, the HIV prevalence among the key populations remains higher than that of the general population. Compared to the national prevalence of 3.1% [11], the HIV prevalence is 3.4% for PWID, 19.4% for brothel based female sex workers, 8.6% for non-brothel based female sex workers, and 22.9% for MSM [12]. Key populations and their sexual partners are estimated to account for about 40% of new HIV infections in Nigeria, although they constitute only 3.4% of the national population [13].

Despite the significant contribution of key populations to the HIV epidemic in Nigeria, there is very little evidence-based information on the drivers of HIV infections for FSW, PWID and MSM in Nigeria. FSW, PWID and MSM are often managed as a homogenous group with an assumption that their HIV sexual risk profile is similar. The absence of evidence to suggest differences in the HIV sexual risk profile of FSW, PWID and MSM makes it difficult to develop specific programs that may reduce the HIV risk of each group. When evidence is provided, the contents of education programs can be developed, which should help individuals to improve their perception of threat and increase the likelihood for behavior change [14]. Behavior change happens when there is a raised consciousness of threat from HIV infection, the individual substitutes risk behaviors with alternative behaviors, reinforces the new positive behavior due to the rewards received, and remove cues that reinforce the negative behavior [15].

The objective of this study was to determine and compare the sexual behavior and HIV sexual risk profiles of FSW, MSM and PWID; and identify factors associated with recent condom use among the groups, with the aim of providing evidence to strengthen HIV interventions for FSW, MSM and PWID in Nigeria.

## Method

### Study design, study sites and study populations

A cross-sectional survey was undertaken in four of Nigeria’s 36 + 1 states – Enugu (South-East Zone), Nassarawa (North-central Zone), Benue (North-central zone), and Akwa-Ibom (South-south zone). These states are four of the eight states supported by the Global Fund for AIDS, Tuberculosis and Malaria to implement HIV prevention programs for key populations. Organized groups of key populations and their members were readily accessible in these states.

Study participants were recruited between April and June, 2015, from a minimum of two different parts of each state where mapping had revealed clusters of MSM, FSW and PWID [9]. A minimum sample of 500 study participants was required (125 participants per state). The target was to recruit 25 FSW, 25 MSM and 25 PWID per each of the two sites in each of the four states. A sample size of 500 was also considered sufficient to facilitate sub-population analysis. All participants were required to be18 years of age or older and to provide written consent for study participation.

### Data collection tool

A semi-structured interviewer-administered questionnaire developed in English, was used for data collection (Additional file 1). Questions were adapted from available instruments for the National HIV, AIDS and Reproductive Health Survey (NARHS). While it might be useful to translate questionnaires into local languages, we found this impractical given the multiplicity of languages in Nigeria. Instead, key words and phrases, especially sensitive ones, were translated to the languages of each selected community and collated in a list generated during the training of interviewers. Interviewers used this list as a reference material in the field. A similar technique was used for National HIV and Reproductive Health Surveys [16–18], the Integrated Biological and Behavioural Surveillance Surveys conducted in Nigeria [12, 19] and the studies by Folayan et al. [20, 21]. Interviewers who understood the local language and had a history of work relationship with target population, were recruited as field workers and trained for this project.

Information on socio-demographics (age, gender, marital status hereby referred to heterosexual conjugal relationships), history of alcohol consumption, use of other psychoactive substances, sexual practices (vaginal, oral and anal sexual intercourse), and high sexual risk behavior for HIV infection (age of sexual debut, consistent use of condom in the last 30 days, and consistent use of condom for anal sex in the last 12 months) were collected. Participants were asked if they used condom every time, almost every time or sometimes when they had sexual intercourse. Consistent condom use was defined as use of condom for every penetrative sexual intercourse. Other responses were classified as inconsistent condom use.

### Study procedure and data collection processes

One focal person for MSM, FSW and PWID respectively and one field worker for each of the target states, had a 3 days training on the data collection process. They also pilot tested the instrument and made recommendations for revisions prior to its printing. The field workers recruited for the survey had had prior experience conducting national surveys. The focal persons were recognized peer leaders for the target communities.

Each population focal person engaged with peers in each of the states, assisted with community entry and community mobilization. The focal person organized a one-day sensitization programme about the research for key peer leaders prior to commencement of participants’ recruitment. The field worker debriefed the peer leaders about the study objectives and methodology and solicited their support to facilitate community entry for the fieldwork. The state focal person helped with study participants’ recruitment (identification of non-governmental organizations working with the key populations, booking of appointments for interview, identification of key populations’ hot spots).

Study participants were recruited through snow balling [22]. This is a method appropriate for finding hidden and hard to reach populations [23] like those recruited for this study. In each state, an initial study participant (the seed) was recruited for the study at each site. This seed was requested to recruit three peers in the first wave of recruitment. The three peers recruited in the first wave of recruitment were requested to recruit three peers each in the second wave of recruitment. The nine peers in the second wave of recruitment were requested to recruit two peers each in the fourth and final wave of recruitment. This way, 31 persons for each target population were anticipated to be recruited per site (2 sites per state). With a fall out rate of 20%, the minimum sample size of 25 persons per site would still be achieved. This procedure helped ensure a broad array of study participants were recruited for the study.

All interviews were conducted in spaces identified as safe and private by state peer leaders. Privacy was ensured and all participants were assured of confidentiality. All study participants signed an informed consent form, were provided with an educational material on HIV prevention and treatment, condoms and lubricants, and were paid N2, 000 ($11.50) for transport reimbursement.

### Data analyses

The proportion of respondents who engaged in each sexual practice (anal, vaginal, and or oral sex), consumed alcohol, used psychoactive substances and engaged in high risk sexual behaviors for HIV infection (inconsistent condom use, early sexual debut) were determined. The prevalence of anal sex was calculated using the check question ‘used condom with sex partner during anal sex in the last 12 months’ with response option: ‘yes’, ‘no’ and ‘never had anal sex’. All respondent who chose ‘yes’ and ‘no’ responses were categorized as having had a history of anal sex.

The association between sexual practices, alcohol consumption, use of psychoactive substances, high-risk sexual behaviors for HIV infection and type of population (FSW, MSM, PWID) was determined. The differences in high-risk sexual behaviors for HIV infection and sexual practices of MSM, FSW and PWID were analyzed using relevant statistical tests: Pearson chi-square was used for categorical variables, and t-test for continuous variables.

Logistic regression analysis was conducted to determine factors associated with consistent use of condom during the last 30 days of the study. The period of recall was also limited to the last 30 days to limit recall bias. The independent variables included in the logistic model were study populations (MSM, FSW, PWID), sexual practice (anal, vagina and oral sex), marital status (married, not married, separated, divorced), alcohol consumption (drink or did not drink alcohol), use of psychoactive drugs (exclusive of alcohol), and ability to negotiate condom use (defined as ability to refuse to have sex when partner refuses to use condom). Statistical significance was determined using p-level (*p* < 0.05) and the 95% confidence interval of the adjusted odds ratio (AOR). Analysis was conducted using STATA version 12.0.

## Results

The questionnaires retrieved from 488 respondents were analysed – response rate of 97.6%. The study population consisted of 188 (38.5%) FSW, 145 (29.7%) MSM and 155 (31.8%) PWID. Six (1.2%) respondents were transgender (Table 1). The key populations groups differed significantly in their marital status: FSW had higher proportion of divorced, widowed and separated persons but less proportion of those who were never married when compared with MSM and PWID (*p* < 0.001).

**Table 1:** Distribution of respondents by background characteristics (*N* = 488)

Variables	Female Sex Worker (FSW) *n* = 188		Men who have Sex with Men (MSM) *n* = 145		Person Who Inject Drugs (PWID) *n* = 155		TOTAL *N* = 488		*P* value
	N	%	n	%	n	%	N	%	
Gender									
Male	0	0.0	139	95.9	137	88.4	276	56.6	< 0.001
Female	188	100.0	0	0.0	18	11.6	206	42.2	
Transgender	0.0	0.0	6	4.1	0	0.0	6	1.2	
State									
Akwa-Ibom	43	22.9	33	22.8	49	31.6	125	25.6	0.002
Benue	67	35.6	29	20.0	29	18.7	125	25.6	
Enugu	37	19.7	46	31.7	39	25.2	122	25.0	
Nasarawa	41	21.8	37	25.5	38	24.5	116	23.8	
Marital Status									
Currently Married	12	6.4	13	9.0	27	17.4	52	10.7	< 0.001
Divorced	20	10.6	0	0.0	4	2.6	24	4.9	
Widowed	26	13.8	1	0.7	1	0.6	28	5.7	
Separated	43	22.9	1	0.7	5	3.2	49	10.0	
Never married	71	37.8	104	71.7	102	65.8	277	56.8	
Others	16	8.5	26	17.9	16	10.3	58	11.9	

### Sexual behaviour

As shown in Table 2, two-thirds or more of each population (71.9%) had their first sexual intercourse as adolescents (10–19 years). There was however no significant difference in age of first sexual intercourse by study population (*p* = 0.16).

**Table 2:** Distribution of all respondents by sexual behaviour and practices (*N* = 488)

Variables	Female Sex Worker (FSW) *n* = 188		Men who have Sex with Men (MSM) *n* = 145		Person Who Inject Drugs (PWID) *n* = 155		TOTAL *N* = 488		*P* values
	N	%	N	%	N	%	N	%	
Forms of sexual intercourse^a^									
Vaginal	187	99.5	75	51.7	150	96.8	412	84.4	< 0.001
Anal	111	59.0	143	98.6	138	89.0	392	80.3	< 0.001
Oral	38	20.2	41	28.3	39	25.2	118	24.2	0.20
Age of first sexual intercourse									
0-9 years	1	0.5	3	2.1	8	5.2	12	2.5	0.16
10–19 years	138	73.4	103	71.5	107	69.0	348	71.9	
20-29 years	24	13.0	24	16.7	24	15.5	72	14.9	
30 years and above	1	0.5	1	0.7	0	0.0	2	0.4	
Cannot remember	21	11.2	13	9.0	16	10.3	50	10.2	
History of use of condom during sexual intercourse									
Ever used condom	186	98.9	140	96.6	148	95.5	474	97.1	0.08
Always used condom during sex in the last 30 days	181	92.3	127	87.6	106	68.4	414	84.8	< 0.001
^b^Always used condom with sex partner during anal sex in the last 12 months	65	34.6	131	90.3	39	25.2	235	48.1	< 0.001
Used lubricant with condom during sex in the last 1 month	141	75.0	112	77.2	43	27.7	296	60.7	< 0.001
Challenges with finding and buying lubricants	67	35.6	39	26.9	43	27.7	149	30.5	0.29
Action when sexual partner refuses to use a condom									
Allow him or her to have sex	20	10.6	29	20.0	48	30.9	97	19.8	< 0.001
Refuse to have sex	141	75.0	75	51.7	76	49.0	292	59.8	

^a^Multiple response

^b^Denominator is number of respondents who reported anal sex

The most common sexual practice by respondents was vaginal intercourse (84.4%) followed by anal sex (80.3%) and oral sex (24.3%). More than half of the MSM respondents (51.7%) practiced vaginal sex. A high proportion of PWID (89%) and FSW (59%) practiced anal sex. The proportion of those who practiced vaginal (*p* < 0.001) and anal (p < 0.001) sex differed among the three populations (FSW, MSM and PWID): vaginal sex was least practiced by MSM while anal sex was least practiced by FSW.

### Use of condom and lubricant

Table 2 shows the distribution of respondents who use condom and lubricants. Almost all respondents (98.3%) had ever used a condom, while 414 (88.4%) reported using condom in the last 30 days. The proportion of respondents who used condom during anal sex in the last 12 months was significantly lower for FSW and PWID when compared with MSM (p < 0.001). More FSW reported using condom during sex in the last 30 days when compared to MSM and PWID (p < 0.001). The lowest proportion condom users were PWID. Significantly more FSW noted that they refuse to have sex if their sexual partners also refuse to use condoms when compared with MSM and PWID (*P* < 0.001).

Only 149 (30.5%) of respondents had challenges finding and buying lubricants. There was no significant difference in the proportion of FSW, MSM and PWID who had challenges finding and buying lubricants (*p* = 0.29).

### Use of psychoactive substance

Table 3 highlights the distribution of all respondents by use of psychoactive substances. The most commonly used psychoactive substance by PWID, MSM and FSW was marijuana followed by codeine and then cocaine. More than a tenth of FSW (17.6%) and MSM (20.7%) had used psychoactive substances. Of those who has used psychoactive substances, more than a quarter of FSW (26.2%) and MSM (29.4%) had injected drugs in the last 30 days of the survey.

**Table 3:** Distribution of all respondents by use of psychoactive substances (*N* = 488)

Variables	Female Sex Worker (FSW) *n* = 188		Men who have Sex with Men (MSM) *n* = 145		Person who Inject Drugs (PWID) *n* = 155	
	n	%	N	%	N	%
Drank alcohol in the last 4 weeks						
Everyday	53	29.1	23	16.0	53	36.1
At least once a week	31	17.0	24	16.7	21	14.3
Occasionally	46	25.3	50	34.7	57	38.8
Do not take alcohol	52	28.6	47	32.6	16	10.9
No Response	6	3.2	1	0.7	8	5.2
History of use of psychoactive substances						
Ever used psychoactive substance	33	17.6	30	20.7	155	100.0
Used drugs in the last 30 days	12	8.8	10	7.4	114	83.8
^a^Forms of psychoactive substance used (by those with history of psychoactive substance use)						
Cocaine	13	29.5	7	20.6	70	45.2
Heroine	3	6.8	4	11.8	41	26.5
Marijuana	23	52.3	18	52.9	134	86.5
Glue	1	2.3	0	0.0	15	9.68
Pethidine	2	4.5	0	0.0	9	5.80
Pentazocine	1	2.3	3	8.82	24	15.5
Chinese capsule	3	6.8	0	0.0	19	12.3
Amphetamines	3	6.8	1	2.94	10	6.45
Rochi	15	34.1	3	8.82	72	46.5
Codeine	15	34.1	8	23.5	94	60.6
Tramadol	11	25.0	3	8.82	59	38.1
Others	1	2.3	0	0.0	12	7.74
Injected drugs in last 12 months	12	27.3	10	29.4	118	76.1
Injected drugs in last 30 days	11	25.0	10	29.4	109	70.3

^a^44 (23.4%) FSW, 34 (23.5%) MSM and 155 PWID gave a history of use of psychoactive substances

Most of the respondents who use psychoactive drugs (65.0%) initiated drug injection when they were 20 years old and above, and three (2.1%) of the 140 respondents who had injected drugs in the last 12 months initiated injecting drug use by age 9 years. Also, 55(39.3%) respondents reported sharing needles and syringes (Table 4).

**Table 4:** History of respondents who Injected drugs in last 12 months (*N* = 140)

Age of injecting drugs initiation^a^		
0-9 years	3	2.1
10-19 years	41	29.3
20-29 years	48	34.3
30 and above	43	30.7
History of syringe and needle sharing		
Every time	8	5.7
Almost every time	7	5.0
Sometimes	40	28.6
Never	52	37.1

^a^We report only the number of respondents who gave a response

### Factors associated with the use of condom in the last 30 days

Table 5 shows the outcome of the logistic regression analysis for factors associated with consistent use of condom in the last 30 days of the survey. The significant factors associated with consistent condom use were use of psychoactive substance, ability to negotiate condom use and the study population. The odds of using condom was significantly lower for MSM (AOR: 0.17; 95% CI: 0.05–0.67; *p* = 0.01) and PWID (AOR: 0.07; 95% CI: 0.02–0.21; *p* < 0.001) when compared with FSW; and for participants who identified they were not able to negotiate condom (AOR: 0.39; 95% CI: 0.18–0.84; *p* = 0.02) compared with those who identified they could negotiate condom use. It was significantly higher for study participants who used psychoactive substances when compared to those who do not use psychoactive substances (AOR: 5.01; 95% CI: 2.59–9.68; *p* < 0.001).

**Table 5:** Logistic Regression analysis on use of condom in the last 30 days (*N* = 488)

Variables			Use condom in the last 30 days		Adjusted odds ratio	Confidence interval	*p* value
			Yes n (%)	No n (%)			
Population	FSW		181 (97.8)	4 (2.2)	1	–	–
	MSM		127 (88.8)	16 (11.2)	0.17	0.05–0.67	0.01
	PWID		106 (76.8)	32 (23.0)	0.07	0.02–0.21	< 0.001
Sexual practice	Vaginal	Yes	350 (84.5)	45 (86.5)	0.96	0.40–3.55	0.76
		No	64 (15.5)	7 (13.5)	1	–	–
	Anal	Yes	143 (34.5)	16 (30.8)	0.90	0.37–2.71	0.99
		No	271 (65.5)	36 (69.2)	1	–	–
	Oral	Yes	98 (23.7)	13 (25.0)	1.17	0.35–1.70	0.51
		No	316 (76.3)	39 (75.0)	1	–	–
Marital status	Currently Married		42 (10.1)	9 (17.3)	1.04	0.44–2.46 –	0.93
	Not currently married		372 (89.9)	43 (82.7)	1	–	–
Partner ask for condomless sex	Agree		72 (17.6	21 (40.4)	2.04	1.06–3.93	0.03
	Disagree		261 (63.8)	22 (42.3)	1	–	–
Alcohol use	Take alcohol		307 (76.4)	39 (75.0)	0.49	0.22–1.09	0.08
	Do not take alcohol		95 (23.6)	13(.0)	1	–	–
Psychoactive substance use	Use		155 (79.9)	39 (20.1)	5.013	2.593–9.684	< 0.001
	Do not use		259 (95.2)	13 (4.8)	1	–	–

Although there was no significant difference in the use of condom in the last 30 days of the survey by sexual practice (vaginal, anal and oral), marital status and history of alcohol use, the odds was higher for those who were currently married when compared with those who are not married, and those who practiced oral sex when compared with those who practice vaginal sex. On the other hand, the odds was lower for those who practice anal sex compared with those who practice vaginal sex, and for those who drink alcohol compared to those who do not drink alcohol.

## Discussion

A high proportion of FSW, MSM and PWID engage in high risk behaviors: they practiced unprotected anal sex; and will not use condom if their partner refuses to use condom; MSM and PWID were less likely to have used condom when compared to FSW; and a lower proportion of FSW and PWID used condom during anal sex when compared with MSM. The proportion of MSM and FSW who use psychoactive drugs and who inject drugs was also high. The use of psychoactive substance and the ability to negotiate condom use were associated with consistent use of condom use in the last 30 days of the survey. Finally, we found that PWID had the highest HIV risk behavior: they were less likely to use condom during sexual intercourse and refuse sex with partners who refused to use condoms when compared with FSW and MSM; and more than a third of the population share needles and syringes.

One of the strength of this study was the engagement of focal persons from each of the key populations with the data collection process. Although they are formally less qualified than the research staff and field workers, the focal persons had extensive experience and understanding of the nuances of their communities [24, 25], which proved valuable in planning and implementing the study. Their engagement enhanced access and responsiveness of peers to the survey questions, strengthened consent and information processes since potential participants felt freer to ask questions [24], and increases the authenticity of the data generated for this study. Their involvement also increased the ability to recruit from different sub-groups of key populations. The extensive training provided to the focal persons on data collection using ethically appropriate methods and techniques, enhanced the value added to the research process. A second strength of the research methodology was the recruitment of study participants through four waves of snowballing. This enhanced the diversity of the study participants thereby addressing possible concerns about the representativeness of the sample [26].

The study however had some limitations. The cross-sectional approach limits the study to only establishing associations as no cause-inferential deductions can be made. Also, participants had to recall information on condom use which introduces recall bias. It is also possible that subgroups of the study population not represented by the seeds recruited for the study, may have been inadvertently left out in the recruitment process. Individuals who are hiding due to legal and policy restrictions - FSW, MSM and PWID are criminalized in Nigeria –may also have been left out during the recruitment [27].

We, acknowledge that the use of audio computer assisted self-administered instruments (ACASI) may have improved the reporting on some high-risk behavior. Adebajo et al. [28] had reported improved reporting of high-risk behavior by MSM and PWID in Nigeria with the use of ACASI. In the absence of ACASI, the engagement of peers as community recruiters was the next best option we had and used. The engagement of the focal persons in studies involving key populations in environments with high level of stigma against them like Nigeria, is invaluable irrespective of the use of ACASI.

The study findings provide valuable evidence on peculiar sexual and reproductive health needs of FSW, MSM and PWID in Nigeria. Although a number of studies conducted in Nigeria have examined the sexual pattern and or HIV risk profile of MSM [29–31], FSW [32, 33] and PWID [28], including the bisexual profile of MSM in Nigeria [34], studies comparing findings on sexual and reproductive health of the populations are rarely reported. We are not aware of any study conducted in Nigeria on the study topic prior to our study.

One of the key findings was that PWID had more risk behaviors for HIV infection than MSM and FSW. Ironically, the national HIV prevalence for key populations in Nigeria is lowest for PWID: less than half the prevalence for MSM and FSW; and close to the HIV prevalence for the general population [12]. The reason for this is not clear. Past studies had attributed the low prevalence of HIV infection among PWID to low prevalence of needle sharing [35], but the outcome of this study does not suggest that the prevalence of needle sharing is low. The high vulnerability of PWID to HIV infection identified in this study, and the actual national HIV prevalence figures for PWID suggests that HIV prevention efforts targeting PWID need to be considerably strengthened to keep the HIV prevalence in the community low. Stigma associated with drug use and the criminalization of injecting drug use hinder PWID from access HIV prevention services [36]. Unfortunately, stigmatization and criminalization of drug use are still major problems in Nigeria [37] and may likely detract PWID from accessing HIV prevention services. Currently, the Nigeria HIV prevention programs for PWID do not include needle and syringe exchange and other harm reduction interventions. HIV prevention interventions for PWID also need to include access to treatment for substance abuse since those treated for substance abuse are more likely to adopt safer behaviors [36].

Unlike Lancaster et al. [38], who demonstrated that use of psychoactive substances is significantly associated with lower use of condom during sexual intercourse, we found that users of psychoactive substances were more likely to have used condom in the last 30 days of the survey. The use of psychoactive substances is expected to increase the risk for HIV infection [39]. We assume this finding may not be unconnected to the intense HIV education provided to drug users in the states were the study was conducted. The finding may suggest that the investment of GFATM funds for education of key populations may be having impsct. The finding however need further investigations.

We found that a significant proportion of FSW and PWID practice anal sex. Previous studies conducted in Nigeria had also highlighted that anal sex is practiced between heterosexuals [40–42]. Unfortunately, education about the risk of contracting HIV through anal sex has been limited to the HIV education curriculum of MSM, and may explain the observed higher use of condom for anal among MSM when compared with FSW and PWID. This study finding highlights the need for the national HIV response program to broaden the HIV preventive education to include anal sex education for FSW and PWID.

Psychoactive substance use prevention education and interventions also need to be targeted at MSM and FSW. Prior studies had indicated that a significant number of MSM and FSW inject drugs [38, 43]. This finding further highlights the challenges associated with the design and implementation of siloed programs that may be ineffective in reaching out to populations that have multiple cross-silo-cutting HIV risk behaviors [44]. Our study however did not explore the proportion of MSM and PWID who sell sex. The exchange of sex for money by female PWID was identified as the major driver of the high prevalence of HIV among female PWID [5], and the justification for focusing on sexual prevention of HIV for PWID [5]. Future studies on sexual and reproductive health of key populations in Nigeria could explore transaction sex as a possible third intersect in the HIV risk profile of key populations.

The low proportion of MSM and FSW who use condom consistently in this study; and the global decrease in condom use [45] remains a challenge for HIV prevention among key populations. The continued challenge with use of condom in this study population makes it imperative to explore the use of pre-exposure prophylaxis (PrEP) for key populations who engage in condomless sex. Thus, when screening for persons who should be eligible for PrEP access, the inability to negotiate condom use and the use of psychoactive substance should be an eligibility criteria for MSM, FSW and PWID. Though a national policy on the use of PrEP identified persons at substantial risk of HIV infection eligible for PrEP access [46], and a PrEP demonstration study was conducted with sero-discordant couples in Nigeria [47], there is currently no roadmap drawn on how PrEP can be accessed in Nigeria. With the available evidence on the efficacy of PrEP for those at substantial risk for HIV infection [48], it is an ethical imperative and a human rights requirement that PrEP be made accessible to key populations in Nigeria in view of their HIV risk profile.

## Conclusion

Our study findings indicate that the sexual risk behavior and HIV risk profile of FSW, MSM and PWID are not clearly distinct. There are commonalties in HIV infection risk behaviors amongst the populations – inconsistent condom use during sex, use of psychoactive substances and injecting drug use. The cross-silo-cutting HIV risk behavior we have demonstrated in this study indicates however, that programmatic interventions for the groups cannot be limited to a specific group; interventions to reduce risk of sexual transmission of HIV, and transmission of HIV infection through sharing of needle and syringes are applicable to MSM, FSW and PWID. Future studies need to explore the prevalence of transactional sex among MSM and PWID.

## Additional file

Additional file 1: Data collection tool for sexual practices, sexual behavior and HIV risk profile of key populations in Nigeria (*N* = 488). (RTF 2339 kb)

## Data Availability

Study related data and materials are accessible on request from the lead author.

## Abbreviations

FSW: Female Sex Worker
GFATM: Global Funds for AIDS, Tuberculosis and Malaria
HIV: Human Immunodeficiency Virus
LMIC: Low and Middle Income Countries
MSM: Men who have Sex with Men
PrEP: Pre-Exposure Prophylaxis
PWID: People who Inject Drugs
