# Supplementary material for: Sexual practices, sexual behavior and HIV risk profile of key populations in Nigeria
Source: BMC Public Health. 2019 Sep 2;19:1210. doi: 10.1186/s12889-019-7553-z (PMC6721228; doi:10.1186/s12889-019-7553-z)
Supplement: Supplementary file 1 — Data collection tool for sexual practices, sexual behavior and HIV risk profile of key populations in Nigeria (N = 488). (RTF 2339 kb) [file 12889_2019_7553_MOESM1_ESM.rtf]

ENHANCING KEY POPULATION INTERVENTION IN NIGERIA THROUGH CAPACITY DEVELOPMENT

(EKPIN) PROJECT: ENVIRONMENTAL SCANNING


PEER LED HIV INTERVENTION QUESTIONNAIRE


COLLEGE OF HEALTH SCIENCES

Obafemi Awolowo University

Ile-Ife, Nigeria


1


IDENTIFICATION, ELIGIBILITY, AND CONSENT

	001 STATE ID |__|__|	002 QUEST. ID No. |__|__|__|		
						
	003	Interviewer's Code  [____|____]	004	Interviewer's Name:		
						
						
	005	Date of interview (dd/mm/yyyy)	___ ___ / ___ ___ / ___ ___ ___ ___		
						

Hello. My name is _______________. My colleagues and I are working on this project which is a collaboration between Obafemi Awolowo University, Ile‐ Ife and Heartland Alliance, an organization involved with helping to prevent the spread of HIV infection. We are here to ask you some questions related to Peer – Led HIV interventions in your community and to seek your opinion on how to make such interventions work best in your community to improve HIV/AIDS prevention, treatment, care and support and ensure you are continually in good and sound health. We will appreciate your sincere responses. This would take us approximately 35 minutes or less to fill. We do re‐assure you that the information received will be treated in utmost confidence. The data gathered can never be traced back to you as information on your name nor address will NOT be collected and the interview will not be recorded. However, your participation is completely voluntary and you do not have to answer any questions you do want to. Not participating will have no negative implications for you.

	007	Do I have your agreement to participate?	.............................................YES	1				
			NO	2		2 ➔ STOP		
			...........................................					
		Signature of interviewer certifying that						
			Signature:					
		informed consent has been given by						
			----------------------------------------------------------		
		respondent			
	008	Time at start of interview	___ ___ : ___ ____	| AM | PM	|			
								

9	Checked by Supervisor: Supervisor's Code |___|___| Date ___________ 

Signature 

	010	Data Entry Clerk Code	|___|___|		
					
					

Target groups

(Please circle the appropriate target group that this PARTICULAR questionnaire is being administered to)

No.	Questions and filters	Group Codes	
001	Female sex worker (FSW)	111	
	Man who have sex with other men (MSM)	222	
	Person who inject drug ( PWID)	333	
			


2

SECTION 1: BACKGROUND CHARACTERISTICS

	No.	Questions and filters	Coding categories		Skip to		
	Q100		Time interview started		TIME  [__|__:__|__] AM/PM				
	Q101		RECORD SEX OF THE	1	= MALE					
			RESPONDENT	2	= FEMALE		[__]			
				3=TRANSGENDER					
	Q112			0	= No Religion						
			What is your religion?	1	= Christianity					
			CIRCLE ONE	2	= Islam					
				3	= Traditional		[__]			
				4	= Other [Specify]					
				………………………					
				9	= No Response					

SECTION 2: MARRIAGE AND PARTNERSHIPS

	No.	Questions and filters		Coding categories	Skip to		
	Q201		Which of these categories best	1=Currently Married		If 2,3,4,5 go		
			describes your marital status now?	2=Divorced				
			[READ OPTIONS OUT]	3=Widowed	[___]	to Q203		
				4=Separated				
				5= Never married				
				6	= Others (specify)________				
	Q202		[ASK THOSE WHO ARE	1	= currently married and living				
				with spouse				
			CURRENTLY MARRIED]	2	= currently married but living with		Go to Q301		
				other sexual partner	[__]			
			Are you ……	3	= currently married but not living				
				with spouse or any other sexual				
				partner				
			[ASK THOSE WHO ARE NOT	1	= not married, living with				
	Q203		CURRENTLY OR NEVER	sexual partner				
			MARRIED]	2	= not married, not living with	[__]			
				sexual partner				
			Are you ……	9	= No Response				
									


3

SECTION 3: SEXUAL HISTORY: NUMBER AND TYPES OF PARTNERS

Emphasize confidentiality

	No.	Questions and filters		Coding categories	Skip to		
	Q301		Have you ever had sexual intercourse?	1	= Yes					
				2	= No		[__]	If No,		
			[For the purposes of this survey, “sexual	9	= No Response			go to Q401		
			intercourse,” is defined as vaginal or							
			anal sex.]							
	Q301a		At what age was your first sexual		Age in years  [___|___]				
			intercourse?		Can't remember…….. 88				
									
					No Response………99				
	Q301b		What was the reason for the first sexual		In love………… …....	1				
			intercourse?		Having fun ………………	2				
					Peer pressure……….3				
					To obtain money…………	4				
					Forced ………………  5				
					Others [________________]				
					No response……….9				
	Q302		Have you had sexual intercourse in the	1=Yes					
			last 12 months?	2= No		[__|			
				9=No response					
	Q302a		Which forms of sexual intercourse have	1=Vagina					
			you ever had?	2= Anal					
			Vaginal	3= oral		[__|			
			Anal							
			Oral							
				1	= Yes			If No go to		
	Q303		Have you ever heard of condom?	2	= No			Q307		
				7	= Don't Remember		[__|			
				9	= No Response					
	Q304			1	= Yes			If No go to		
			Have you ever used a condom?	2	= No					
				7	= Don't Remember		[__|	Q307		
				9	= No Response					
	Q305		Have you ever used a condom with any	1	= Yes					
				2	= No					
			of your sex partners in the last 3 months?	7	= Don't Remember					
				9	= No Response					
										


	Q306		READ OUT: Please take time to think about your answer to these				
			questions so that we can get the most accurate information possible.				
			Remember we are not recording your name and this information is strictly				
			confidential.					
			Think about the sexual partners you've had in the last 12 months?				
			(MULTIPLE ANSWERS POSSIBLE)					
			How many were:					
			-   A. Your spouse(s) or live-in sexual partners	REGULAR			
								
					[__|__]__]			
					888 = Don't Know		
			- B. Your “boyfriend” or 
C.  “girlfriend”		999 = No Response		
								
					BOY/GIRL FRIEND		
					[__|__]__]			
					888 = Don't Know		
			-   C. partners with whom you had sex in exchange for gifts or money
D. Did you receive monies or gifts?
Did you receive monies or gift?	999 = No Response		
							
					COMMERCIAL		
					[__|__]__]			
					888 = Don't Know		
			-   D. Sexual partners to whom you are not married, have never lived	999 = No Response		
							
			with, are not your boyfriend or girlfriend and you did not pay for sex?	Casual			
					[__|__]__]			
					888 = Don't Know		
					999 = No Response		
								
		
		
		
			sex?					
	Q308		Have you ever used a condom with any of	1= Yes				
				2= No				
			your sex partners during anal sex in the last	7= Don't Remember	[__]			
			3 months?	8= Don't Know				
								
				9= No Response				
	Q308a			1= To prevent pregnancy				
			What was the MAIN reason you or your	2= To prevent STI				
			partner used a condom the last time you had	3= To prevent HIV				
			Anal sex?	4= Partner insisted				
				5= Don't trust partner				
			RECORD THE MAIN REASON	6= Just for the fun of it	|__|__|			
			MENTIONED	7= It's convenient				
				8= Others				
				……………………….				
				88 = Don't Know				
				99 = No Response				
	Q309		Have you ever used a lubricant with any of	1= Yes				
				2= No		àIf		
			your sex partners during anal sex in the last	7= Don't Remember				
			3 months?	8= Don't Know	[__]	no,		
				9= No Response		go to		
								
						Q310		
							
	Q309b		If yes, please can you specify the type of					
			lubricant you used during your last sexual					
			intercourse [write the name of the lubricant					
			used]					
	Q310		Have you used condom in the last one	1= Yes				
			month	2= No	[__]			
				9= No Response				
						5		

		Q310a		How often did you use condoms in the last 3 months?	1= Every time			
					2= Almost every time			
					3= Sometimes			
					4= No response			
		Q311			1= Yes			
				Do you apply lubricants when using	2= No	[__|		
				condom in the last one month?	9= No Response			
		Q311a			1= Every time			
				How often do you apply lubricants when	2= Almost every time			
				using condom?	3= Sometimes	[__|		
					4= No response			
		Q312		Do you know of any place or person from	1= Yes			
				which you can obtain male condoms?	2= No	[__|		
					9 = No Response			
		Q312a		Do you have challenges with finding and	1= Yes			
				buying male condom?	2= No	[__|		
					9 = No Response			
		Q313		Do you know of any place or person from	1= Yes			
				which you can obtain female condoms?	2= No	[__|		
					9 = No Response			
		Q313a		Do you have challenges with finding and	1= Yes			
				buying female condom?	2= No	[__|		
					9 = No Response			
		If yes, why do you have challenges?		Not Available……….A	
	Q313b	[read out options)		Too Expensive……….B	
				Partner Objected……….C	
				Don't Like Them……….D	
			Used Other Contraceptive………E	
			Didn't think it was necessary………  F	
				Didn't Think Of It……….G	
				Don't know condoms…..H	
			Others specify[	]….I	
				No Response…………….J	
	Q314	Do you have challenges with finding and	1= Yes				
		buying lubricants?	2= No		[__|		
			9 = No Response			
	Q314b	If yes, why do you have challenges?		Not	Available……….A	
				Too Expensive……….B	
				Partner Objected……….C	
				Don't Like Them……….D	
			Used Other Contraceptive……….E	
				Didn't Think It Was……....F	
				Necessary……….G	
				Didn't Think Of It……….H	
				Don't know condoms…..I	
			Others specify[	]….J	
				No Response…………….K	
		What will you do if your spouse or live-in	1=Allow him/ her to have sex		
	Q315	sexual partner refuses to use a condom, and	2=Examine him/her before sex		
		you want him/her to do so?	3=Refuse to have sex completely	[__|	
			4= Others, specify ……....		
	Q316	In the last 3 months, how many sexual	1 - One				
		partners have you had?	2- More than one		
			Specific number |__|__|__|		
			88 = Don't Know		
			99 = No Response		
							


6

SECTION 4. SOCIAL HABITS (ALCOHOL/DRUG USE) [ALL GROUPS]

Q401		1	= Every day				
	During the last 3months, how often would	2	= At least once a week				
	you say you drank alcohol?	3	= Occasionally	[__|			
	..READ OUT	4	= Never				
		99 = No Response	[__|			
Q402		Cocaine (Crack, chunk)		àIf		
	Some people have tried a range of	Heroine	[__|			
	different types of drugs. Which of the	Marijuana (Weed)	[__|	none,		
	following, if any, have you ever tried to	Glue	[__|	go to		
	get high?	Pethidine	[__|	Q501		
	READ LIST	Pentazocine (Fortwin)	[__|			
		Chinese Capsules	[__|			
	MULTIPLE RESPONSE POSSIBLE	Amphetamines	[__|			
		Rochi (Rohypnol)	[__|			
		Codeine	[__|			
		Tramadol (Tramal)	[__|			
		Other drugs (Specify)____	[__|			
		None	[__]			
Q403	Some people have tried injecting drugs	No response	[__]	àIf no,		
							
	using a needle and syringe. Have you	1= Yes		go to		
	injected drugs in the last 12 months?	2	= No	[__|	Q409		
	DRUGS INJECTED FOR MEDICAL	8	= Don't Know				
	PURPOSES OR TREATMENT OF	99 = No Response				
	AN ILLNESS DO NOT COUNT						

	Q404		Which of the following types of drugs	LIST A “USED”						
										
			have you used in the past one month?	Used in last month						
			READ LIST A for “USED”.							
			MULTIPLE ANSWERS POSSIBLE.	YES  NO  DK	NR			
								
			Pentazocine (Fortwin)	1	2	8	99	[__|		
			Heroin (not in combination with cocaine)	1	2	8	99	[__|		
			Cocaine (not in combination with heroin)	1	2	8	99	[__|		
			Heroin and cocaine together	1	2	8	99	[__|		
			Cocaine (crack, chunk)	1	2	8	99	[__|		
			Pethidine	1	2	8	99	[__|		
			Marijuana (Weed)	1	2	8	99	[__|		
			Pentazocine (Fortwin)	1	2	8	99	[__|		
			Chinese Capsules	1	2	8	99	[__|		
			Amphetamines	1	2	8	99	[__|		
			Rochi (Rohypnol)	1	2	8	99	[__|		
			Codeine	1	2	8	99	[__|		
			Tramadol (Tramal)	1	2	8	99 [__|		
			Other drugs (Specify)____	1	2	8	99	[__|		
			Anything else? (specify)	1	2	8	99	[__|		
										


7

Q405	Which of these drugs have you injected	LIST B “INJECTED”					
	in the past one month?	Injected in last month						
	READ LIST B” for INJECTED” .							
	MULTIPLE ANSWERS POSSIBLE.	YES  NO  DK	NR			
						
	Pentazocine (Fortwin)	1	2	8	99	[__|		
	Heroin (not in combination with cocaine)	1	2	8	99	[__|		
	Cocaine (not in combination with heroin)	1	2	8	99	[__|		
	Heroin and cocaine together	1	2	8	99	[__|		
	Crack	1	2	8	99	[__|		
	Pethidine	1	2	8	99	[__|		
	Marijuana (Weed)	1	2	8	99	[__|		
	Pentazocine (Fortwin)	1	2	8	99	[__|		
	Chinese Capsules	1	2	8	99	[__|		
	Amphetamines	1	2	8	99	[__|		
	Rochi (Rohypnol)	1	2	8	99	[__|		
	Codeine	1	2	8	99	[__|		
	Tramadol (Tramal)	1	2	8	99	[__|		
	Other drugs (Specify)____	1	2	8	99	[__|		
	Anything else? (specify	1	2	8	99	[__|		
FOR PEOPLE WHO INJECT DRUGS ONLY							
No.	Questions and filters	Coding categories			Skip to		
406	How long have you been injecting drugs?	Number Of Months		[__[__|__]			
		Record 000 If Less Than 1 Month				
		888 = Don't Know						
407		9999 = No Response						
	How old were you when you first injected	Age In Completed Years	[__|__]			
	addictive/non-medical drugs? (Includes	Don't Remember	77					
	self-injection or injection by another).	Don't Know	88					
	[Estimate Best Answer]	No Response	99					
	Think about the times you have injected	Estimate Best Answer						
		1 = Every Time						
408	drugs during the past one month. How	2 = Almost Every Time						
	often did you use a needle or syringe that	3 = Sometimes		[__|			
	had previously been used by someone	4 = Never						
		8 = Don't Know						
	else?							
		99 = No Response						
409								
	Are you aware of any HIV & AIDS	1 = Yes		[__|			
	prevention programs in this city aimed at	2 = No					
	drug users?	99 = No Response						


8

SECTION 5. PERCEPTION ABOUT THE SERVICE PROVIDED BY PEER LED GROUPS

Once again, remember that the information you provide is strictly confidential. However, the information would be found useful in making plans for HIV programming for the community. If you recall, I informed you about Heartland Alliance and their plans to try and or support existing organisations to provide needed HIV prevention, treatment, care and support services for community members. I will like to ask you questions about how you think this project would work.

501. What are the factors that you think may make services provided by peers organisations acceptable?

	Factors	Yes	No	Don't know	No response	
		1	2	3	4	
501a	Ability to pay for services					
501b	The distance of service delivery points to home	
501c	Lack of knowledge about HIV by providers	
501d	Lack of friendly providers	
501e	Inadequate Information specific to MSM	
501f	Inability to provide HIV counselling services	
501g	Stigmatization by providers	
501h	Inability to manage stigma related crisis	
501i	Inability to address police harassment	
501j	OTHERS(PLEASE SPECIFY)	
502	How do you think these challenges identified above (Including those you identified) can the addressed?	


SECTION 6. I WILL LIKE TO KNOW ABOUT YOUR CONCERNS ON HOW THE CHALLENGES IDENTIFIED IN QUESTION 502 CAN AFFECT THE FOLLOWING SERVICES LISTED BELOW EXPECTED TO BE PROVIDED TO KEY POPULATIONS AS YOURS

Kindly tick the figures representing each of the following challenges below against the listed services. You can tick as many challenges that you think may apply

Negative Peer Relationships ……………...1	Concern about sustainability……………4	
Stigmatization……………………………. 2	Fear of interference by Police …………. 5		
Cultural Hostility………………………….3	Lack of funding ………………………....6		
     
PREVENTION SERVICES							
601	MSM	1	2	3	4	5	6	
601a	Community based outreaches							
601b	Community HIV counseling and testing							
601c	Using role models for peer led HIV education							
601d	Community based STI prevention, screening and treatment							
601e	Peer led distribution of condoms							
601f	Peer led distribution of lubricants							


9

CARE AND SUPPORT SERVICES

611	MSM	1	2	3	4	5	6	
611a	
611b	poverty alleviation	 	 	 	 	 	 	
611c	Formal education	 	 	 	 	 	 	
611d	Vocational education	 	 	 	 	 	 	
611e	Access to other health promoting services e.g. psychosocial counseling	 	 	 	 	 	 	
611f	Peer support group	 	 	 	 	 	 	
611g	Support services to adherence to ART	 	 	 	 	 	 	
611h	Legal support services	 	 	 	 	 	 	


10

SECTION 7. USE OF PEER LED ORGANIZATIONAL ACTIVITIES

		perceptions of MSM on the need for HIV prevention services							
									
												
		How willing are you to:		Very	Willing	Neutral	Un-	Very			
				willing			Willing	unwilling				
												
			1	2	3	4	5				
											

701	Attend regular meetings to attend HIV related issues?	[__]		
702	Allow peers to ask you questions about your risk	[__]		
703	behaviour?	[__]		
	Follow a plan with you to help you address your			
704	HIV risk?			
	How willing are you to receive and condoms?	[__]		
705	How willing to receive and use lubricants?	[__]		
706	Visit clinics for STI check-ups by peers?	[__]		
707	How willing are you are to go to Public health facilities for HIV related services ?	[__]		
708				
	Attend clinics if accompanied by peers?	[__]		
709	How willing peers facilitate access to services in public			
710	hospitals if you encounter difficulties			
	How willing to access HCT every three month?	[__]		
711	Have peers serve as drug adherence supporter if	[__]		
	HIV positive?
How willing to have your peers working			


How willing are you to receive legal services to address discrimination on your sexual orientation

How willing are you to receive justice for discrimination based on your sexual orientation


11

Which of the under-listed services have you received in the past from any peer led groups, association, organisation (Please tick one)

Yes	No	Don't know	No response	
1	2	8	9	

		801			Information on Family planning					[__|			
													
													
		802			Family planning services					[__|			
													
													
		803			Information/education about STI					[__|			
													
													
		804			Syndromic management of STI					[__|			
													
													
		805			Safe sex promotion					[__|			
													
													
		806			Men involvement in reproductive health					[__|			
													
													
		807			Male condom promotion/distribution					[__|			
													
													
		808			Female condom promotion/distribution					[__|			
													
													
		809			Lubricant promotion/distribution					[__|			
													
													
		810			HIV screening in ANC					[__|			
													
													
		811			Couple HIV counselling and testing					[__|			
													
													
		812			Support for HIV positive mothers					[__|			
													
													
		813			Maternal and newborn care					[__|			
													
													
		814			Prevention of unsafe abortion					[__|			
													
													
		815			Post abortion care					[__|			
													
													
		816			Prevention of gender based violence					[__|			
													
													
	817			Others not mentioned above include:								
												
												
		818			Kindly mention the names of such existing peer-led organisations you	know offering the above listed services to			
					such key populations as yours in your community.						
													
					Names of Known Peer- led organizations / associations/ facilities:						
													


12

SECTION 9. CLIENT SATISFACTION WITH PUBLIC HEALTH SERVICES.

The following questions are to assess your level of satisfaction with the services offered by these public (government) organizations / associations / facilities (particularly the HIV related services)

NO	QUESTIONS	RESPONSES  AND CODING	SKIP TO		
Once again, thanks for the time given to provide responses to the questions so far. I still have a few more questions.		
Please, try to cast your mind back to the services you have received from public hospital and give responses to the		
following questions below.				
					
901	Have you received HIV and sexual and	1= Yes	Go to Q1001		
	reproductive health services before from a public	2= No			
	health service?	9 = No Response			
902	If yes, how satisfied are you with the extent to	Very dissatisfied……………1			
					
	which service providers listen to your problems	Dissatisfied….………………2			
	and concerns?	Neutral………………………3			
		Satisfied…………………….4			
		Very Satisfied………………5			
		No Response……………….99			
					
903	How satisfied are you with the extent to which	Very dissatisfied……………1			
					
	service providers assure you of confidentiality	Dissatisfied….………………2			
	and privacy?	Neutral………………………3			
		Satisfied…………………….4			
		Very Satisfied………………5			
		No Response……………….99			
					
904	How satisfied are you with the extent to which	Very dissatisfied……………1			
					
	service providers respect your rights as a service	Dissatisfied….………………2			
	recipient?	Neutral………………………3			
		Satisfied…………………….4			
		Very Satisfied………………5			
		No Response……………….99			
					
905		Distance to services……………1			
					
	What barriers have you encountered in accessing	Cost…………………………….2			
	the services provided by these peer- led	Confidentiality ………………...3			
	organizations?	Waiting time …………………..4			
	[Circle all that apply.]	Hours of operation ……………5			
		Staff attitudes …………………6			
		Other: ………………………….7			
		No Barriers…………………….8			
					
906	Would you consider looking for another public	Yes ……………1			
					
	hospital/organization / association from which	No …………….2			
	you can access these Reproductive Health & HIV	Do not know….88			
	related services?	No response …..99			
					
907	Please tell me why you want to seek for another	hospital/organization / association to access these HIV related		
				
	services?				
					


13
